# The correlation between GFR and unit renal volume in infants with hydronephrosis measured by two imaging methods

**DOI:** 10.1038/s41598-023-46996-y

**Published:** 2023-11-09

**Authors:** Ke Guo, Deshan Zhao

**Affiliations:** Department of Nuclear Medicine, The Second Hospital of Shanxi Medical University, No.382 Wuyi Road, Taiyuan, 030001 Shanxi Province China

**Keywords:** Kidney diseases, Paediatric research, Kidney diseases, Urology, Paediatric urology

## Abstract

The purpose of this study was to investigate the correlation between GFR and unit renal volume in infants with congenital hydronephrosis measured by ^99m^Tc-DMSA static renal imaging and ultrasonography. According to the classification of hydronephrosis, 38 infants aged 0–12 months with congenital hydronephrosis were divided into six groups: healthy kidney groups, mild hydronephrosis groups, and severe hydronephrosis groups. Within one week, all patients underwent ultrasound, diuretic dynamic renal imaging, static renal imaging and lateral imagings of both kidneys after static renal imaging respectively. Pediatric renal volume was calculated using the improved formula length × width × thickness × 0.674, and then the renal function in per unit volume (GFR/unit volume, ml/cm^3^) was obtained. All statistical analysis was done with SPSS Statistics version 24.0. The renal function in per unit volume was a minimum of 1.62 ml/cm^3^ in left healthy kidney in static renal imaging, but the renal function in per unit volume was a maximum value of 2.20 ml/cm^3^ in right healthy kidney in ultrasonography. There was a strong positive correlation observed between GFR and renal volume in left healthy kidney group and left and right kidneys with mild hydronephrosis groups (r = 0.865, r = 0.872, r = 0.822). A moderate positive correlation was found between GFR and renal volume in right healthy kidney group and left and right kidneys wih severe hydronephrosis groups (r = 0.783, r = 0.542, r = 0.798). GFR in per unit volume ranged from 1.62 to 2.20 ml/cm^3^ in healthy kidney, and was significantly higher in right kidney as compared to in left kidney, and also decreased with the progression of hydronephrosis.

## Introduction

The congenital hydronephrosis in children is mainly caused by ureteropelvic junction obstruction (UPJO)^1^. In general, the majority of fetuses will gradually improve after experiencing short hydronephrosis, a minority of fetuses still retain hydronephrosis up to 1 year after childbirth^2^. The progression or improvement of hydronephrosis can be determined by monitoring the changes in affected kidney function. The purpose of this study is to help infants with congenital hydronephrosis who are less than 12 months old to reduce their pain and medical cost during seeing the doctors, and strive to find a simple and effective examination method that can basically achieve the diagnostic and follow-up goals. Hope to effectively solve the problem through looking for a fixed correspondence between kidney function and unit renal volume. Due to the fact that the patients in the study is in the infant period and a large number of nephrons in kidneys have not yet fully maturity, the correlation between renal function and renal volume is basically in a relatively stable state. Given that the remaining renal parenchymal tissues in kidneys with severe hydronephrosis have the properties similar to normal renal tissues, but this relationship can not exist in adult patients with hydronephrosis. Therefore, although the treatment intervention for infants with hydronephrosis is based on the change of GFR, if the remaining renal parenchymal tissue in kidneys can basically conform to the normal nature of kidney tissue, it means that a certain renal volume can roughly reflect the corresponding kidney function. Ultrasonography technique, a simple and effective examination method, can be used to follow up and repeatedly observe the changes of affected kidney function and hydronephrosis in infants in the short term (such as 1–3 months), due to its advantages of non-invasiveness, convenient operation and easy for coordination of children^3^. However, CT and diuretic dynamic renal imaging have been limited to varying degrees in clinical applications for infants with hydronephrosis due to factors such as ionizing radiation or cumbersome technical procedures. Therefore, the research of the corresponding relationship between renal function and unit volume in single kidney has a important clinical value for understanding and predicting the change of affected kidney function in patients with moderate or severe hydronephrosis. The correlation between GFR estimated by ^99m^Tc-DTPA diuretic dynamic renal imaging and unit renal volume measured by ultrasonography and ^99m^Tc-DMSA static renal imaging will be explored in this study.

## Materials and methods

### General material

A prospective analysis was conducted on 38 infants with congenital hydronephrosis (including 11 neonates) who underwent ultrasonography at The Shanxi Children's Hospital and ^99m^Tc-DMSA static renal imaging and ^99m^Tc-DTPA diuretic dynamic renal imaging at The Second Hospital of Shanxi Medical University. The patient cohort consisted of 31 males and 7 females, with an average age of 4.37 ± 3.48 months (age range: from 23 days to 12 months), an average height of 64.97 ± 8.85 cm (height range: from 51 to 80 cm), and an average weight of 7.38 ± 2.46 kg (weight range :from 3.3 kg to 11 kg). These three examinations were completed within a time interval ranging from three to seven days. According to multidisciplinary consensus on the classification of prenatal and postnatal urinary tract dilation (UTD classification system) in 2014, UTD grading system in prenatal fetuses and postnatal infants can be divided into four types^4^: normal, mild, moderate and severe hydronephrosis. Thirty eight infants with congenital hydronephrosis were divided into six groups: healthy kidneys groups, mild hydronephrosis groups, and severe hydronephrosis groups. These categories are equivalent to specific UTD risk stratification categories: normal, UTD A1 (low risk), UTD A2-3 (increased risk); UTD P1 (low risk), UTD P2 (intermediate risk), or UTD P3 (high risk). The study included a total of 76 kidneys: 28 healthy kidneys (10 left kidneys and 18 right kidneys), 22 kidneys with mild hydronephrosis (12 left kidneys and 10 right kidneys), 5 kidneys with moderate hydronephrosis (2 left kidneys and 3 right kidneys), and 21 kidneys with severe hydronephrosis (14 left kidneys and 7 right kidney). Exclusion criteria for abnormal kidney cases were solitary kidney, duplicated kidney, polycystic kidney disease, or ectopic kidney. Informed consent was obtained from all patients involved in the study. All methods were conducted following relevant guidelines and regulations. This study has been approved by the Ethics Committee of the Second Hospital of Shanxi Medical University under petition number (2022) YX No. (122).

### Imaging instruments and radiopharmaceuticals

Sonographic imaging was performed with a ultrasound (Volusion E8 Expert, General Electric Company, NY, USA). Diuretic dynamic renal imaging and static renal imaging were conducted using a dual-head gamma camera (SPECT, Discovery NM/CT 670, General Electric Company, NY, USA) equipped with a low energy high-resolution collimator set at a 20% window and 140 keV photopeak. Pentetate (DTPA) and dimercaptosuccinic acid (DMSA) were supplied by Beijing Xinkesida Medical Technology Co., Ltd. Beijing, China. Tc-99 m was provided by Atom High-Tech Co. Ltd. Beijing, China. ^99m^Tc-DTPA and ^99m^Tc-DMSA were labeled and used within 1 h. The labeling efficiency of ^99m^Tc-DTPA and ^99m^Tc-DMSA exceeded 95%.

### Diuretic dynamic renal imaging

Adequate hydration was ensured in infants by feeding 5 ml of water or breast milk per kilogram of body weight over a period of 30 to 60 min prior to the study^5^. Sedation should be induced in infants through rectal administration of a solution containing 10% chloral hydrate. Patients should be positioned supine during the procedure. Following intravenous bolus injection of approximately 53.68 ± 8.29 MBq (range: 43.29–78.81 MBq) of ^99m^Tc-DTPA, acquisition of image data would be enabled in posterior view using a matrix size of 64 × 64 at frame rates of 30 frames/2 s and 180 frames/10 s, a dose of 1 mg/kg furosemide was administered intravenously around 15 min into the study.

### Static renal imaging

^99m^Tc-DMSA static renal imaging was performed within 1⁓3 days after diuretic dynamic renal imaging. ^99m^Tc-DMSA was administered intravenously with a dose of 3.7 MBq/kg (average dose: 56.94 ± 10.48 MBq, range: 45.51–70.67 MBq) and a volume of 0.3⁓0.5 ml. Patient was examined in the supine position, and posterior, anterior views (700 kcounts/view), left and right lateral views (300 kcounts/view) in a 128 × 128 matrix format at 1 to 2 h after the injection of ^99m^Tc-DMSA^7^.

### Image data processing and glomerular filtration rate measurement

#### Renal volume measurement

**Figure 1:**
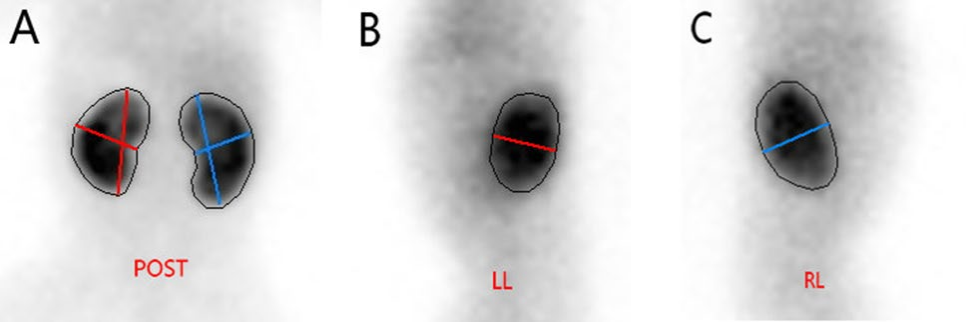
The method of measuring the length, width and thickness of kidneys on static renal imaging. (**A**) The length and width of both kidneys were measured on posterior static renal imaging. (**B**) The thickness of left kidney were measured on left lateral view of static renal imaging. (**C**) The thickness of right kidney were measured on right lateral view of static renal imaging. a, b and c represent the length, width and thickness of kidneys respectively. POST: posterior view. LL: left lateral view; RL: right lateral view.

The maximum length and width of the kidney on the coronal plane in ultrasonography and static renal imaging, respectively, should be measured. The maximum length is measured from the lower pole to the upper pole of the kidney, while the maximum width is measured from the anterior to the posterior margin of the kidney at the hilum on the coronal plane of the kidney^8^. Additionally, the thickness of the kidney is measured from its anterior to posterior margin on either right or left lateral view (Fig. 1). After obtaining patient data for the length, width, and thickness of the kidneys using two imaging methods, these values are entered into a adjustment formula for calculating renal volume in children: length × width × thickness × 0.674^9^, and calculating the renal volumes separately for each imaging method.

#### GFR measurement

Regions of interest were manually drawn over both kidneys as well as an area inferior and lateral to their lower poles for background subtraction on a composite image taken between 2 and 3 min using Xeleris image processing system equipped with SPECT/CT produced by GE company. After inputting patient data regarding weight and height into Gates algorithm, single kidney GFR (GFRsingle) as well as GFR for both kidneys were automatically calculated^10^.

### Statistical analysis

All statistical analysis was done with SPSS Statistics version 24.0. Paired t-test was used for analyzing quantitative data. All values were expressed as the mean ± standard deviation (SD). Pearson correlation analysis was conducted between GFR and renal volume of single kidney within six groups along with obtaining respective regression equations. A correlation coefficient ׀r׀ ≥ 0.8 indicates high correlation; 0.5 ≤ ׀r׀ < 0.8 denotes moderate correlation; 0.3 ≤ ׀r׀ < 1 signifies low correlation; ׀r׀ < 0.3 suggests weak correlation. *P* < 0.05 indicates a statistically significant difference.

## Results

### Comparisons of kidney length, width and volume between ultrasonography and static renal imaging

The length, width and volume of both kidneys in healthy kidneys and mild hydronephrosis groups measured by static renal imaging were slightly larger than that from ultrasound. Conversely, the length, width and volume of both kidneys in severe hydronephrosis groups were slightly smaller than that from ultrasound. Significant differences were observed between the length, width and volume values of both kidneys across all groups using two methods (*p* < 0.05) (Table 1).

**Table 1:** Comparisons of the length, width of kidneys and renal volume in infants with congenital hydronephrosis measured by static renal imaging and ultrasound (${\overline{\text{x}}}$ ± s).

HN Classification	Weight (kg)	Numbers	Ultrasonography			Static renal imaging		
			Length	Width	Renal volume	Length	Width	Renal volume
Normal								
L Kid	3.9 ± 3.14	10	5.73 ± 0.43	2.56 ± 0.31	27.17 ± 5.60	5.86 ± 0.35*	2.66 ± 0.27#	29.63 ± 5.42†
R Kid	5.39 ± 3.57	18	5.64 ± 0.84	2.51 ± 0.14	23.17 ± 4.70	5.79 ± 0.81*	2.62 ± 0.14#	25.96 ± 4.56†
HN								
L mild	5.42 ± 4.08	12	6.18 ± 0.98	2.85 ± 0.37	31.47 ± 7.14	6.32 ± 0.99*	3.01 ± 0.38#	33.62 ± 6.69†
R mild	2.90 ± 2.56	10	5.99 ± 0.92	2.67 ± 0.37	26.29 ± 4.90	6.17 ± 0.98*	2.84 ± 0.35#	28.90 ± 4.50†
L severe	2.93 ± 1.98	14	7.95 ± 0.68	3.99 ± 0.45	75.98 ± 18.04	7.78 ± 0.66*	3.91 ± 0.41#	73.35 ± 17.74†
R severe	3.25 ± 3.20	7	7.11 ± 0.78	3.56 ± 0.36	62.92 ± 16.45	6.89 ± 0.84*	3.47 ± 0.38#	59.69 ± 16.30†

HN, hydronephrosis; L Kid, left kidney; R Kid, right kidney; L mild, mild hydronephrosis in left kidney; R mild,mild hydronephrosis in right kidney; L severe, severe hydronephrosis in left kidney; R severe, severe hydronephrosis in right kidney;*Comparisons of the length of kidneys in normal groups, mild HN groups and severe HN groups measured by static renal imaging and ultrasound: t(*p*) = 3.55 (0.006), t(*p*) = 7.42 (0.001), t(*p*) = 2.35 (0.039), t(*p*) = 2.71 (0.024), t(*p*) = − 4.46(0.001), t (*p*) = − 2.68(0.025); #Comparisons of the width of kidneys in normal groups, mild HN groups and severe HN groups measured by static renal imaging and ultrasound: t(*p*) = 6.71 (0.001), t(*p*) = 8.09 (0.001), t(*p*) = 4.18 (0.002), t(*p*) = 5.67 (0.001), t(*p*) = − 3.80(0.047), t(*p*) = − 3.29(0.017); †Comparisons of renal volumes in normal groups, mild HN groups and severe HN groups measured by static renal imaging and ultrasound: t(*p*) = 16.82(0.001), t(*p*) = 18.75(0.001), t(*p*) = 3.42(0.006), t(*p*) = 4.91(0.001), t(*p*) = − 3.72(0.003), t(*p*) = − 5.66(0.001); *p* < 0.05 was considered significant statistically.

### Comparisons of renal function in per unit volume between ultrasonography and static renal imaging

The renal function in per unit volume (GFR in single kidney/renal volume, ml/cm^3^) was lower in healthy kidneys and mild hydronephrosis groups when measured by static renal imaging as compared to ultrasound. However, the renal function in per unit volume was higher in severe hydronephrosis groups when measured by static renal imaging compared to ultrasound. The smallest recorded value for renal function in per unit volume in a normally functioning kidney was 1.62 ml/cm^3^ in left healthy kidney using static renal imaging while the largest value was 2.20 ml/cm^3^ in right healthy kidney using ultrasonography. Furthermore, there was a significantly higher level of renal function in per unit volume observed in right kidney compared to left kidney with statistical significance noted across all groups (*p* < 0.05) (Table 2). These findings indicate a gradual decrease in renal function in per unit volume with increasing severity of hydronephrosis.

**Table 2:** The correlation between GFR and renal volume in infants with congenital hydronephrosis measured by static renal imaging and ultrasound (${\overline{\text{x}}}$ ± s).

HN Classification	Numeber	GFR (ml/min/1.73m^2^)	Renal function in per unit volume (ml/cm^3^)		
			Ultrasonography	Static renal imaging	t (*p*)
Normal					
L Kid	10	47.10 ± 4.29	1.76 ± 0.28	1.62 ± 0.21	− 6.95 (0.001)
R Kid	18	50.17 ± 4.70	2.20 ± 0.26	1.95 ± 0.19	− 11.18 (0.001)
HN					
L mild	12	46.43 ± 6.42	1.52 ± 0.20	1.42 ± 0.17	− 4.16 (0.002)
R mild	10	46.51 ± 5.11	1.82 ± 0.22	1.64 ± 0.12	− 3.67 (0.006)
L severe	14	41.78 ± 2.28	0.58 ± 0.15	0.60 ± 0.15	2.44 (0.031)
R severe	7	38.41 ± 2.78	0.62 ± 0.14	0.66 ± 0.17	3.46 (0.018)

HN,hydronephrosis; GFR,glomerular filtration rate; L Kid, left kidney; R Kid, right kidney;L mild, mild hydronephrosis in left kidney; R mild,mild hydronephrosis in right kidney; L severe, severe hydronephrosis in left kidney; R severe, severe hydronephrosis in right kidney; Renal function in per unit volume is GFR in single kidney/renal volume(ml/cm^3^), *p* < 0.05 was considered significant statistically.

### The relationship between GFR and single kidney volume in infants with hydronephrosis measured by ultrasonography

**Figure 2:**
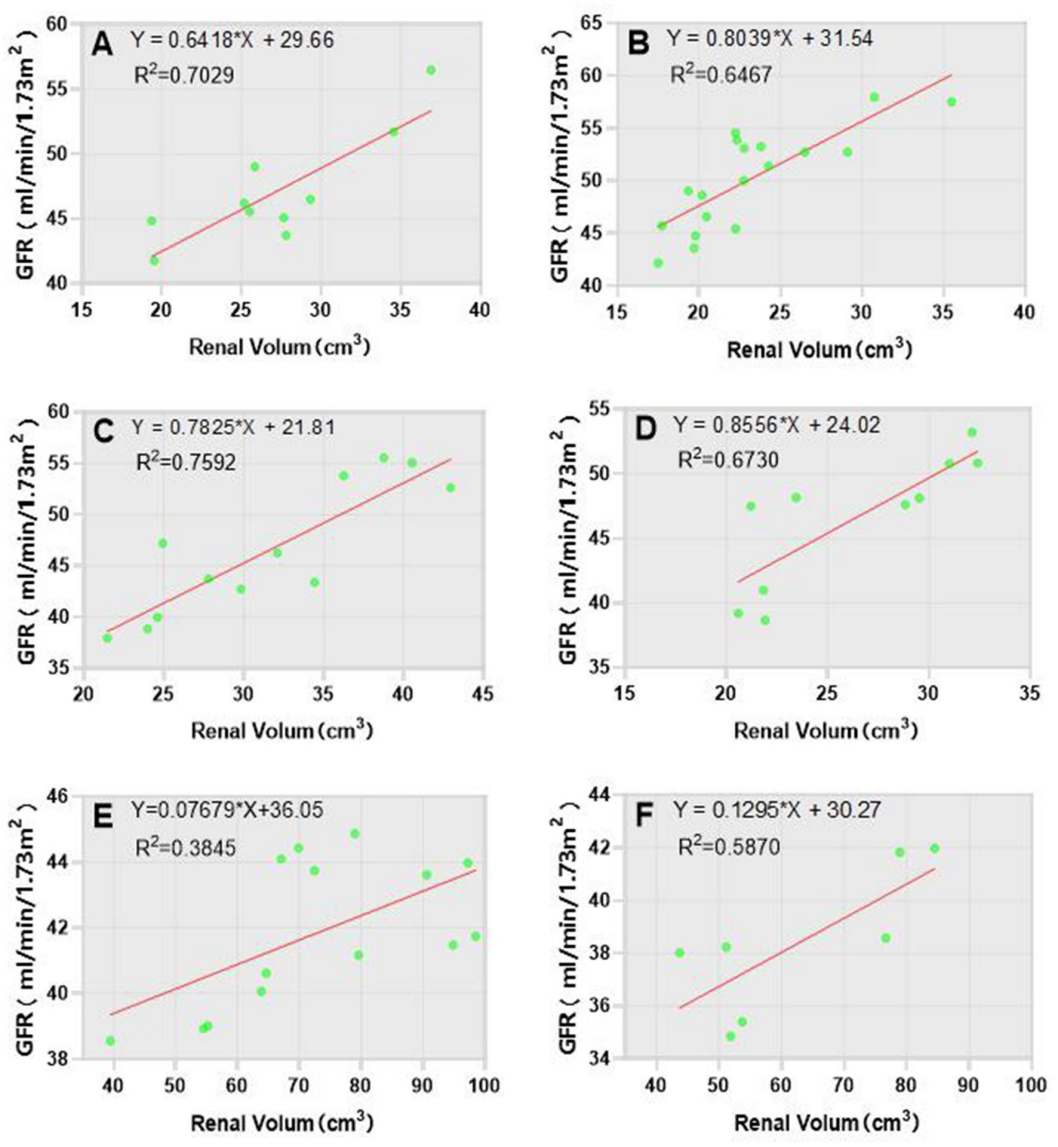
Linear regressions of right and left kidneys in normal, mild and severe hydronephrosis groups.GFR: glomerular filtration rate; *R*^2^: Goodness of fit, is the setting degree of regression line to the observed value. (**A**) Linear regression between GFR and renal volume in normal left kidney group; (**B**) Linear regression between GFR and renal volume in normal right kidney group; (**C**) Linear regression between GFR and renal volume in mild hydronephrosis of left kidney group; (**D**) Linear regression between GFR and renal volume in mild hydronephrosis of right kidney group; (**E**) Linear regression between GFR and renal volume in severe hydronephrosis of left kidney group; Linear regression between GFR and renal volume in severe hydronephrosis of right kidney group.

There was a strong positive correlation observed between GFR and renal volume in left healthy kidney group, as well as in left and right kidneys with mild hydronephrosis groups (r = 0.865, r = 0.872, r = 0.822). In contrast, a moderate positive correlation was found between GFR and renal volume in right healthy kidney group, as well as in left and right kidneys wih severe hydronephrosis groups (r = 0.783, r = 0.542, r = 0.798). Linear relationships were observed between GFR and renal volume measured by ultrasonography within these groups, leading to the derivation of linear regression equations for each respective subgroup (Fig. 2).

## Discussion

The incidence of congenital hydronephrosis in children is approximately 0.13⁓0.16%, with a higher prevalence in boys, and infants account for 25% of cases. The occurrence of congenital hydronephrosis is more commonly seen on the left kidney, which account for up to 2/3 of newborns, while bilateral involvement occurs in about 10–40%^11^. Most cases of pediatric hydronephrosis tend to resolve or stabilize over time due to gradual development of affected kidneys during follow-up. However, a minority may experience worsening symptoms which significantly increase their risk for impaired renal function. The younger these patients are, the more severe their urinary tract obstruction is, and the more severe their hydronephrosis is^2^, resulting in an increase in the degree of affected kidney damage. Hence, closely monitoring the patients with non-physiological hydronephrosis allows early detection followed by timely intervention.

Given that the renal function in infants correlates closely with kidney size, evaluating both size and function of the kidney through measuring kidney length has become a common approach. Moreover, it is possible that assessing renal function based on the correlation between GFR and renal volume holds even greater value^12^. CT examination is considered the gold standard for measuring kidney size, but its use is limited to infants and young children due to ionizing radiation. However, Janki et al.^13^ found that ultrasound measurements of kidney size were nearly equivalent to those obtained by CT. Therefore, ultrasound can be used as an alternative method for measuring kidney size and monitoring changes in certain circumstances due to its advantages such as non-invasiveness, operability, and relative accuracy in size measurement, however, it cannot estimate renal function. To address this limitation, renal volume can be estimated using a adjustment formula that incorporates the length, width, and thickness of the kidneys measured by ultrasound in children. The clinical value of ultrasound can be further enhanced when combined with GFR measured by diuretic dynamic renal imaging to calculate renal function in per unit volume. This approach allows for monitoring changes in renal function and hydronephrosis and early detection of urinary tract obstructive hydronephrosis in infants. Nevertheless, ultrasonography-based measured renal volume may be influenced by factors such as the tangent plane used on the kidney surface or the region being measured or sonographer’s subjective experience. Similarly, ultrasonography-based measured length of the kidney is also different at the waist compared to at the back of body^14^. Because there are some limitations associated with ultrasound including more subjectivity and lack of strict objectivity or stable quantitative data, in this study static renal imaging was employed as a control method for measuring renal volume due to its advantages such as high counting rate^15^, clear images of the kidneys, low statistical error, and better repeatability of length, width, and thickness measurements. In this study, the commonly used ellipsoid formula (length × width × thickness × π/6^16^) was not employed for estimating renal volume due to its tendency to underestimate^17^. Instead, a more accurate adjustment formula proposed by Zakhari (length × width × thickness × 0.674^9^) was adopted.

The kidneys were divided into six groups based on the severity of unilateral hydronephrosis: healthy kidney groups, mild hydronephrosis groups, and severe hydronephrosis groups in infants with hydronephrosis. However, the kidney with moderate hydronephrosis was excluded from the study due to a limited sample size (5 kidneys; 2 left and 3 right). Renal function in per unit volume was obtained using ultrasound and static renal imaging. The results demonstrated that the length, width, and volume of both left and right kidneys in healthy kidneys groups and mild hydronephrosis groups were slightly larger when measured by static renal imaging compared to ultrasound. Conversely, renal function in per unit volume was lower when measured by static renal imaging than by ultrasound. In contrast, for severe hydronephrosis groups, the length, width, and volume of both kidneys were slightly smaller when measured by static renal imaging compared to ultrasound; however, renal function in per unit volume was higher when measured by static renal imaging than by ultrasound. These differences among all groups were statistically significant. Nevertheless, both methods consistently showed a gradual decrease in renal function in per unit volume with increasing severity of hydronephrosis. This finding aligns with previous studies indicating progressive impairment of renal function associated with varying degrees of hydronephrosis.

The smallest renal function in per unit volume in a kidney with normal function was 1.62 ml/cm^3^ (left kidney) in static renal imaging, while the largest renal function in per unit volume in a kidney with normal function was 2.20 ml/cm^3^ (right kidney) in ultrasonography. Furthermore, the renal function in per unit volume was significantly higher in right kidney compared to left kidney. Additionally, it existed a linear relationship between GFR and renal volume measured by ultrasonography within groups, from which linear regression equations were derived. These findings demonstrate that under different disease conditions, the functional capacity of kidneys varies corresponding to their respective renal volumes^18^. Moreover, these regression equations within groups facilitate observation and monitoring of changes in both renal volume and renal function in per unit volume in unilateral hydronephrotic kidneys over time using ultrasound. By utilizing this corresponding relationship, we can preliminarily estimate changes in hydronephrotic kidney function based on alterations observed in the parenchymal volume of ultrasound images and promptly detect rapidly increasing hydronephrotic volumes as well as deteriorating kidney functions; thus providing an opportunity for timely diuretic dynamic renal imaging and surgical intervention.

## Data Availability

The datasets generated and analysed during the current study are not publicly available due to privacy but are available from the corresponding author on reasonable request.
